# I Approve of the Organization of a Veterinary Contingent to the Army

**Published:** 1899-03

**Authors:** Russell A. Alger

**Affiliations:** Secretary of War


THE JOURNAL

OF

COMPARATIVE MEDICINE AND

VETERINARY ARCHIVES.

VOL. XX. *MARCH, 1899.* No. 3.

### "I APPROVE OF THE ORGANIZATION OF A VETERINARY CONTINGENT TO THE ARMY.

"(Signed) RUSSELL A. ALGER,
SECRETARY OF WAR."

MR. ALGER is the first Secretary of War who has indorsed and aided the veterinary profession in its attempts to obtain a proper recognition and legislation for its rights.

The Hull Bill (H. R. 11022) in the recent Congress, provided "two veterinarians" for each regiment of cavalry, as follows:

The veterinarians provided for in this section shall have the rank, pay, and allowances of a second lieutenant of cavalry: *Provided*, That the veterinarians provided for above shall stand a competitive examination to be held under rules and regulations to be prescribed by the Surgeon-General of the United States: *And provided further*, That the veterinary surgeons now in service with cavalry regiments may be appointed as veterinarians without reference to age: *And provided further*, That in computing length of service for retirement such veterinarians shall be credited with the time they have already served as veterinary surgeons.

The Senate sub-committee replaced this section, which was accepted by the Republican half of the Senate committee of military affairs, with the following:

Of the veterinarians provided for in this Act one shall have the pay and allowances of a second lieutenant of cavalry, and one with the pay of seventy-five dollars per month and the allowances of a sergeant-major: *Provided*, That the veterinarian appointed to the first grade shall not be so appointed until he shall have passed an examination, to be prescribed by the Secretary of War, as to his mental, moral, and professional qualifications: *Provided further*, That the veterinarians now in the service who do not pass such competitive examination shall be eligible to the positions of the second class under such rules as are now prescribed by the regulations. The regimental sergeant-major and the regimental quartermaster-sergeant provided for in this section shall have the pay and allowance of an ordnance sergeant.

It was then evident that something more energetic must be done, and appeal was made to the President and the Secretary of War. The President without hesitation said that he would approve of the organization of a veterinary corps for the army. The Secretary of War, Russell A. Alger, promptly indorsed in writing an amendment with these words : "*I approve of the organization of a veterinary contingent to the army.*"

The amendment presented by Senator Kenney, of Delaware, to the Army Bill was :

[From Congressional Record, February 27, 1899.]

Mr. Kenney: Mr. President, I desire to send to the desk and have read for the information of the Senate some amendments that I shall propose to the pending bill.

The presiding officer (Mr. Chilton in the chair): The amendments will be read.

The Secretary read as follows:

Amendments intended to be proposed by Mr. Kenney to the bill (H. R. 11022) for the reorganization of the Army of the United States and for other purposes, viz.:

On page 1, in line 11, after the word "corps," insert the following, "a veterinary corps."

On page 2, in line 13, strike out the words "two veterinarians."

On page 3 strike out all after the word "privates," in line 7, down to and including the word "regulations," in line 19.

On page 7, at the end of section 7, add the following:

"Sec. —. That the veterinary corps shall consist of a chief veterinary officer, with the rank, pay, and allowances of a colonel, United States Army; 10 veterinarians, each with the rank, pay, and allowances of a first lieutenant of cavalry; and 20 veterinarians, each with the rank, pay, and allowances of a second lieutenant of cavalry.

"The original vacancies in the force created by this section may be filled by selections from veterinarians who have served in the armies of the United States in the war with Spain or from civil life: *Provided*, That no person shall be appointed a lieutenant in this corps until he shall have passed a satisfactory examination as to his physical, moral, and professional qualifications before a board to be appointed by the Secretary of War, of which board the chief veterinary officer shall be one, whose proceedings and findings shall first be approved by the Secretary of War.

"All rules and regulations governing the veterinary corps shall be made by the Secretary of War, and the chief veterinary officer shall report directly to that officer."

Mr. Kenney then said: Mr. President, I shall trespass upon the time of the Senate as briefly as possible in an endeavor to explain some of the reasons why, in my judgment, the amendments which have just been read should be incorporated into the bill now pending in the Senate.

In any reorganization of the Army of the United States there is no question of more importance to its efficiency and welfare than a properly established veterinary corps. In and out of the army for many years there have been men who have appreciated the value and necessity of a veterinary corps in our army, and on more than one occasion bills looking to that end have been formulated and presented to Congress, but none have ever been enacted into law.

Most of the European nations have as a part of their military establishment well-organized veterinary corps.

The English army has a principal veterinary surgeon with the rank of a colonel, a first-class veterinary surgeon attached to each army corps, a first-class veterinary surgeon attached to the inspector-general of the line of communication, with subordinate grades of veterinary surgeons and veterinary surgeons on probation.

The French army has 5 first-class principal veterinary surgeons with the rank of colonel, 10 second-class principal veterinary surgeons with the rank of lieutenant-colonel and major, 122 first veterinary surgeons with the rank of captain, 232 second veterinary surgeons with the rank of first lieutenant, 91 assistant veterinary surgeons with the rank of sublieutenant.

The German army has an inspector-general of the military veterinary medical service with the rank of colonel of cavalry, to each army corps a veterinary inspector, to each regiment a staff veterinary surgeon, and to every squadron, battery, or train battalion a first- or second-class veterinary surgeon, all commissioned officers.

In the Italian army there is an inspector-general of military veterinary medicine with the rank of colonel, 1 veterinarian with the rank of colonel, and 1 with rank of lieutenant, with inspector-general's office at headquarters of the army; 2 with rank of lieutenant-colonel and 10 with that of major to the 12 army corps as inspectors of public animals; 24 with rank of captain and 48 with rank of lieutenant to the 24 regiments of cavalry; 12 with rank of captain and 24 with rank of lieutenant to the 12 regiments of artillery; 12 with rank of captain and 36 with rank of lieutenant to the battalions of the train; 2 with rank of lieutenant to engineer corps; 1 rank of captain to military school; 1 rank of captain, 2 rank of lieutenant, to cavalry school.

If veterinary service is so important in foreign armies it should not be less valuable in the Army of the United States. If the humane sentiment in the countries mentioned sanctions such an elaborate organization to provide proper care and treatment for animals, should not the United States take an equally advanced position in this respect? In my judgment it should, and no better or more fitting time could be found than now.

The efficiency of an army in the field does and must depend upon the health and strength of its force, and the health and strength of the troop and battery animals are as much to be desired as that of the men. To be certain of the efficiency of army animals is most important.

Of first importance in this regard is that animals purchased for army service should be inspected, before acceptance, by men who are in every way competent to inspect, and the graduate professional veterinarian is the one who alone can and does fill this requirement. Next is the proper care of the health of these animals after they have become the property of the Government, and again there is but one who can be charged with this responsibility—the veterinarian. Not alone are we to be benefited by the incorporation of this corps into our army because of what it will do in the selection and care of animals necessary to its organization, but as well will this corps be most valuable in the inspection of flesh food for our troops.

It is the veterinarian who, by reason of his education and experience, is best able to pass upon the fitness of animal food for our army.

I venture the assertion that had we had a properly and well-organized veterinary corps during the late war with Spain upon which had rested the responsibility for the selection and care of army animals and inspection of meat for our soldiers, many thousands of dollars would have been saved to the Government and the deplorable condition now existing in the War Department would never have been. The necessity for such a corps does to me seem apparent when the value of army animals is taken into consideration. The following figures show the number and cost of army animals to the Government for the periods named:

| | Peace period, from July 1, 1897, to March 31, 1898 (9 months). | | | War period, from April 1, 1898, to August 31, 1898 (5 months). | | |
|---|---|---|---|---|---|---|
| | No. | Total cost. | Average cost. | No. | Total cost. | Average cost. |
| Cavalry horses . . . | 668 | $84,274 50 | $126 15 | 10,743 | $1,078,813 82 | $100 42 |
| Artillery horses . . | 118 | 16,260 00 | 137 79 | 2,551 | 333,807 11 | 130 85 |
| Draft horses . . . . | 39 | 5,776 50 | 148 11 | 1,137 | 142,561 75 | 125 38 |
| Riding horses . . . | 1 | 123 75 | 123 75 | *2,115 | 164,330 00 | 77 70 |
| Bell horses . . . . | ...... | .............. | ......... | 32 | 11,595 00 | 49 84 |
| Pack horses . . . | ...... | .............. | ......... | 40 | 1,200 00 | 30 00 |
| Draft mules . . . | 215 | 21,466 48 | 99 84 | 17,515 | 1,927,608 40 | 110 05 |
| Pack mules . . . | 38 | 2,771 60 | 72 93 | 2,667 | 221,774 00 | 83 15 |
| Total . . . | 1079 | $130,672 83 | ......... | 36,800 | $3,871,690 08 | ......... |

From this statement it will be seen that during the period of five months, viz., from April 1 to September 1, 1898, the cost to the Government for horses and mules for all branches of the service was little short of $4,000,000.

The following extract from the report of the Legislative Committee of the American Veterinary Medical Association I desire to read:

The proper selection and care of the horses, mules, and other animals in the cavalry, artillery, and transportation service and of those taken to be slaughtered for subsistence is a very important consideration, and upon it must depend to a large extent the efficiency and success of the army.

Horses should be selected with a view to proper size and conformation for the service in which they are to be used; they should be free from lameness and other forms of unsoundness, the mares should not be pregnant, and all animals should be specially examined for contagious diseases. These points can only be determined by a competent veterinarian. They would be passed upon most carefully by an official with rank and responsibility, who would be held to a proper accountability for the condition of the animals.

When horses and mules in the service are injured or become lame it is important that they should be skilfully treated. If contagious diseases, such as glanders, break out, the proper measures should be at once taken to prevent the spread of the contagion and to stamp it out. Such measures can be intelligently formulated and enforced by expert veterinarians only.

The provision in the bill for two veterinarians with the rank of second lieutenant mounted for each cavalry regiment makes the position of such veterinarians more agreeable, and will have a tendency to secure better men and to give them some standing and authority. But it should be

* Includes 1500 little horses for Cuban service.

understood that this does not provide an adequate veterinary service. It leaves the artillery, the transportation service, the inspection of public animals, and the military schools without military veterinarians. It also fails to provide veterinary advice for the War Department or the headquarters of the army. Surely there should be at least one veterinarian of greater ability and experience than can be obtained for the pay of a second lieutenant who can advise as to the medicines, instruments, regulations, and orders to be issued to the veterinarians in the several regiments. There should be some one with the most expert knowledge to see that the regulations are carried out and that contagious diseases are properly guarded against and suppressed. The experience of the past summer should be sufficient demonstration of this fact.

In 1890 Dr. R. S. Huidekoper, late chief medical director, First Army Corps, of Philadelphia, Pa., in an address before the students of the veterinary department of the University of Pennsylvania on this subject, said:

In the middle ages we find frequent mention of veterinarians attached to the bodies of troops who bristled at the heels of all the kings and great nobles of Europe. First in Italy and Spain, and later in France and Germany, the necessity of expert care of war-horses was recognized, and we find mention of military veterinarians who were held in much esteem. Under the first Republic in France, Lafosse, who had formerly been veterinarian at the court of the King, was appointed inspector-general of the horses in the cavalry. In the first year after the foundation of the Alfort Veterinary School officers from the armies of Austria, Prussia, and Denmark were enrolled among its students.

In 1769 A.D., each cavalry regiment of the French army furnished one student to the school, and in 1774 the French Government established twenty free scholarships for students who would agree to enter the army after finishing their studies. Later the number was raised to forty. While the veterinary schools in France are in charge of the department of agriculture, the department of war takes an active interest in them, and the army secures about one-half of the graduates each year. The end of the last century and the beginning of the present one saw veterinary schools established in all the countries of Europe, either directly by the government or with government aid, except in England, where the schools have always been private enterprises.

In the more peaceful countries, like Switzerland and Denmark, the schools are attached to the departments of agriculture, but receive support from the departments of war, and furnish in return veterinarians to the army. In France and Prussia the schools have been in turn under the direction of the minister of war and of agriculture. In Prussia the army controls over nine-tenths of all the students. Many of these enlist, and after carrying the musket for one year are sent to the Berlin school in uniform and under pay, where they receive their education at the expense of the government. For each six months at the school the soldier has to serve one year as a veterinarian in the army; so, with the three and a half years required at this school, the service is seven years. If the student fails in one half-year examination, and has to take an extra six months' instruction, he is called on to serve his eighth year in the army before he can retire to the more profitable civil offices and private practice.

In Austria the veterinary school was founded at Vienna by Maria Theresa and completed by the Emperor Joseph II. essentially as a military institution, which it is to-day, under the direction of the department of war. The discipline is strictly military, and the destination of the greater percentage of the students is the army. In Russia and Italy the veterinary schools are classed more closely with the general educational institutions, but both have a majority of military students. In the former country the same system of free education for future military service that exists in Prussia is in vogue. It is only within the last half century that the army veterinarians of Europe have been commissioned officers.

In Prussia the veterinarians enter the army as noncommissioned officers, and after two years receive the commission of lieutenant; but it must be remembered that the army surgeon in the same country only became a commissioned officer in 1845. In all other civilized countries except the United States the veterinarian enters as a lieutenant and may be promoted to the rank of colonel. In France the veterinarians became officers, with a definitely organized corps, in 1843. During the several years previous to this the loss of horses in the French army was from 80 to 85 per 1000; in 1845 it fell to 77 per 1000. In 1852 the service was improved by increase of pay, pension, and retirement, securing better veterinarians, and the annual loss fell to 67 per 1000. In 1860 considerably increased pay again attracted better men, and the loss fell to 28 per 1000.

Dr. Schwartzkopf, in speaking of the present army veterinarians, said:

If we now review the professional position of the veterinary surgeon we find that he has but little support in regard to his duties and privileges. Very little is said about it in the army regulations and cavalry tactics, and this with open cautiousness. This puts the veterinary surgeon in a doubtful place, and his position is dependent by favor or disfavor of his commanding officer and his special orders in regard to veterinary matters. No office or veterinary hospital is given him; he is compelled to go to the different stables for the treatment of the sick animals.

The gathering together and carrying of implements from one stable to another in special cases causes trouble and delay, and much treatment must be omitted by want of proper medicine, instruments, and any arrangement for certain diseases. The veterinary surgeon gets but little willing assistance from the farrier and shoeing smith, who logically should be his subordinates; but he has no authority over them, his instructions are carelessly executed, and his orders in the treatment and shoeing of horses often changed by others in authority, who measure their competence to indulge in the practice of scientific medicine among horses by virtue of their rank, which the veterinary surgeon does not possess.

Now, the responsibility of the troop commander over his horses under treatment of a veterinary surgeon is a great drawback, as it prevents the latter from using his judgment and energies in critical cases, for it is almost certain that a difference of opinion may exist, and all exact knowledge and practice gained by years of studying in colleges and universities goes to naught by reason of prejudice and superstition. But cavalry tactics require of officers that they shall be able to treat all ordinary cases of injury and disease in horses. This is exactly the point in view one hundred years ago, but to-day is an absurdity. The only competent authority to treat disease in horses is the veterinary surgeon, and he alone should be responsible for the proper medical treatment.

But the general rules of the hygienic care of horses in their natural condition, the knowledge of hippology, should be well known to every cavalry officer. To be a good horseman is a necessary qualification of a cavalry officer, and gives him a wide field of studies, as hippology contains the history of the horse, the history of the art of breeding, training, saddling, riding; also the sanitary care and economy of the horses in regard to the principles and practice of stabling, feeding, watering, grooming, and finally the knowledge of the exterior conformation of the horse on a general anatomical basis, with the view of proper judgment for the purchase of horses to the different branches of the service. But quite separate from modern hippology is veterinary medicine and surgery; that is absolutely a medical science of vast extent.

It is a deplorable fact that the United States is far behind European nations in the matter of veterinary surgery. It is the only government in the world that does not make its army veterinarians commissioned officers. This defect must be apparent to every man who has had the slightest understanding of military matters. Contract veterinarians who have no official authority are as helpless as can be in the enforcement of orders.

It has been said to me that the young officers of the army oppose the provision covered by these amendments, for the reason they do not want to be ranked by horse-doctors. Mr. President, does not every well-informed person know that to-day the brightest and most exclusive young men of the country are veterinarians, and that the classes in veterinary surgery of the great universities and colleges of this country have among their numbers young men of the very highest classes in the land? The tramp horse-doctor of half a century ago and the veterinarian of to-day are no more akin than are the doctors of medicine of to-day and the barber blood-letters of centuries ago.

I know of no calling or profession more to be honored and encouraged than that which labors for the care, relief, and improvement of our domestic animals.

No dishonor can attach to anyone by contact or association with such men. The fact is the army will be the better when they are a part thereof.

The present system cannot but bring to the service men of inferior abilities; and while there are many men in the service of much fitness, yet a veterinarian of high class cannot seek government service where his authority can be questioned by the men in the ranks.

Now, therefore, Mr. President, I hope that the amendments just read will be adopted for the reason that if reorganization of the army is to be had no more advanced step can be taken than one which will engraft on our army service a veterinary corps. I hold that the expense of the corps will be paid and money saved by its adoption.

The losses to our government during the last war in horses by reason of a lack of proper medical care would support for years a veterinary corps as provided in these amendments.

In this connection, Mr. President, I desire to submit, and ask to have printed as a part of my remarks, without reading it, a letter from Dr. Leonard Pearson, the dean of the department of veterinary medicine of the University of Pennsylvania.

The Presiding Officer: The letter referred to will be printed, in the absence of objection.

The letter referred to is as follows:

UNIVERSITY OF PENNSYLVANIA,
DEPARTMENT OF VETERINARY MEDICINE,
PHILADELPHIA, February 15, 1899.

DEAR SIR: One of the provisions of the army reorganization bill that has recently been passed by the House of Representatives gives veterinarians in cavalry regiments the rank of second lieutenant.

The office of veterinarian in the United States Army has always been without rank, dignity, authority, or adequate compensation. The position has therefore been of a most humiliating description and one that an educated and self-respecting man could not long endure.

All of the leading civilized nations, including England, Germany, France, Italy, Russia, and Spain, have competent army veterinary departments, and their army veterinarians have commissioned rank or position corresponding thereto. In England the chief veterinarian ranks as colonel; in Russia, as general.

There are now in this country numerous veterinary schools—some independent, some connected with universities, such as Harvard, Cornell, Pennsylvania, Ohio, and Iowa—that prepare veterinarians for the work of their profession as thoroughly as physicians are prepared.

The veterinary profession is now recognized as of importance and dignity, and it is evident that veterinarians must have a good education and full scientific equipment to meet the new and enlarged demands of their calling. There can be no doubt that a properly organized army veterinary service would save several times the amount of its cost annually in the improvement of the quality of remounts and the increased term of usefulness of the army horses, provided the positions are made such as well-trained men can accept without sacrifice of self-respect.

On these grounds may I ask you to support the provision in the army reorganization bill that confers the rank of second lieutenant on veterinarians in cavalry regiments?

Yours, very truly,

LEONARD PEARSON,
Dean.

HON. RICHARD R. KENNEY,
Senate Chamber, Washington, D. C.

Mr. President, I have endeavored to explain as best I could the reasons why these amendments should be engrafted upon the bill now pending before the Senate; but whether agreed to or rejected, nevertheless, to my mind, there seems no reason why the bill now under consideration should not be enacted into law.

A vote for Mr. Kenney's amendment was twice called for by Senator Chandler, of New Hampshire, but the floor was conceded to general discussion, and the amendment was lost in the general business.

The Army Bill passed with the provisions of the Senate sub-committee.

